# Making change last? Exploring the value of sustainability approaches in healthcare: a scoping review

**DOI:** 10.1186/s12961-020-00601-0

**Published:** 2020-10-13

**Authors:** L. Lennox, A. Linwood-Amor, L. Maher, J. Reed

**Affiliations:** 1 National Institute for Health Research, Applied Research Collaboration North West London. Imperial College London, 369 Fulham Road, SW10 9NH London, United Kingdom; 2 Ministry of Health, Environment, Culture and Housing, George Town, Grand Cayman KY1-9000 Cayman Islands; 3 Ko Awatea Health System Innovation and Improvement, Middlemore Hospital, 100 Hospital Road, Otahuhu, New Zealand; 4 Julie Reed Consultancy, 27 Molasses House, London, SW113TN United Kingdom

**Keywords:** Sustainability, tools, frameworks, models, healthcare improvement, sustainability outcomes

## Abstract

**Background:**

Numerous models, tools and frameworks have been produced to improve the sustainability of evidence-based interventions. Due to the vast number available, choosing the most appropriate one is increasingly difficult for researchers and practitioners. To understand the value of such approaches, evidence warranting their use is needed. However, there is limited understanding of how sustainability approaches have been used and how they have impacted research or practice. This review aims to consolidate evidence on the application and impact of sustainability approaches in healthcare settings.

**Methods:**

A systematic scoping review was designed to search for peer-reviewed publications detailing the use of sustainability approaches in practice. A 5-stage framework for scoping reviews directed the search strategy, and quality assessment was performed using the Mixed Method Appraisal Tool. Searches were performed through electronic citation tracking and snowballing of references. Articles were obtained through Web of Science, PubMed and Google Scholar. Six outcome variables for sustainability were explored to ascertain impact of approaches.

**Results:**

This review includes 68 articles demonstrating the application of sustainability approaches in practice. Results show an increase in the use of sustainability approaches in peer-reviewed studies. Approaches have been applied across a range of healthcare settings, including primary, secondary, tertiary and community healthcare. Approaches are used for five main purposes, namely analysis, evaluation, guidance, assessment and planning. Results outline benefits (e.g. improved conceptualisation of sustainability constructs and improved ability to interpret sustainability data) and challenges (e.g. issues with approach constructs and difficulty in application) associated with using a sustainability approach in practice. Few articles (14/68) reported the sustainability outcome variables explored; therefore, the impact of approaches on sustainability remains unclear. Additional sustainability outcome variables reported in retrieved articles are discussed.

**Conclusions:**

This review provides practitioners and researchers with a consolidated evidence base on sustainability approaches. Findings highlight the remaining gaps in the literature and emphasise the need for improved rigour and reporting of sustainability approaches in research studies. To guide future assessment and study of sustainability in healthcare settings an updated list of sustainability outcome variables is proposed.

**Trial Registration:**

This review was registered on the PROSPERO database CRD 42016040081 in June 2016.

## Background

Healthcare research continuously produces new and innovative findings but there is evidence that these improvements are often forgotten, changed or replaced in practice long before they can reach wider populations [1–6]. Therefore, the sustainability of evidence-based interventions has been identified as one of the most critical gaps in implementation science [3, 7, 8].

In healthcare, the term sustainability often refers to broad notions of continuation or permanence over time but the concept of what will be sustained is diverse [1, 9, 10]. While there is currently no consensus in the field on which definition of sustainability to operationalise and advance, for this paper, the comprehensive definition developed by Moore et al. will be used, namely that: “*after a defined period of time, a program, clinical intervention, and/or implementation strategies continue to be delivered and/or individual behaviour change is maintained; the program and individual behaviour change may evolve or adapt while continuing to produce benefits for individuals/systems*” [11]. This definition allows sustainability to be viewed as both an ‘outcome’ where health benefits and intervention activities are maintained, as well as an ongoing ‘process’, recognising the need to respond and adapt to promote continuation of improved practices, benefits or outcomes [12].

Rising demands and competition for resources have highlighted the need to understand if and how the sustainability of implemented initiatives can be influenced to support the long-term impact of investments [3, 4]. Sustaining improved outcomes and processes is a recognised challenge to healthcare staff and stakeholders. Numerous definitions and determinants of sustainability make it exceedingly complicated for researchers and practitioners to understand or influence sustainability in practice [3, 5, 13–16]. To address the challenge of conceptualising, measuring and influencing sustainability, many researchers and healthcare practitioners have developed sustainability frameworks, models and tools to assess sustainability in their initiatives or settings [17–26]. (The term ‘sustainability approaches’ will be used throughout this paper to refer to sustainability models, frameworks, tools, checklists, processes, strategies and conceptualisations [27]).

A 2018 systematic review identified 62 sustainability approaches designed for use in healthcare settings [27]. The review found that sustainability approaches may offer a means of connecting findings across diverse sustainability studies by providing explicit definitions and foundations for measurement but it also concluded that further research was needed to understand the application of these approaches in practice to assess their potential impact on sustainability processes and outcomes [17, 19, 27]. Although there is evidence of mounting interest in using sustainability approaches, little work has been conducted to understand how approaches have been used and how they have impacted research or practice [28]. This has resulted in seemingly random selection of approaches across research studies driven by ‘convenience or prior exposure’ [29–31]. This issue highlights an ongoing gap in knowledge between the development of frameworks, models or tools and the understanding of how they are used in and impact research and practice [29].

In order to understand the value of approaches such as models and frameworks, researchers and practitioners require supporting evidence to warrant their use [32]. Clear descriptions of how approaches inform planning and conduct of sustainability research, along with the potential challenges of use, would provide insight into which approaches have demonstrated positive impact for different programmes and settings [3, 7, 19, 27, 33, 34]. Combining this evidence across sustainability approaches may also support further theory development to create improved frameworks and models for sustainability [19, 35, 36].

In the available literature, it is also not currently known if or how sustainability approaches are contributing to sustainability outcomes or improving sustainability reporting. Proctor et al. defined ‘sustainability outcomes’ as the “*subsequent impact (healthcare improvement or public health outcomes) of sustained intervention use*” [37]*.* To understand the impact of the approaches, more rigorous measurement and conceptualisation of sustainability outcomes is needed for the ‘methodological objectivity and interpretability’ of findings [3, 17]. Scheirer and Dearing suggest six sustainability outcome variables [17], namely (1) continuation of benefits or outcomes for consumers, clients or patients; (2) continuation of programme activities or components of the original intervention; (3) maintenance of partnerships developed during the funded programme; (4) maintenance of new organisational practices, procedures and policies; (5) sustaining attention to the issue or problem; and (6) programme diffusion and replication in other sites.

These variables provide a quantifiable way to understand the success of a project or intervention [17, 36]. Assessing the impact of sustainability approaches on these variables will support a nuanced understanding of how sustainability approaches contribute to sustainability outcomes, aiding researchers and practitioners to critically appraise their value to research and implementation efforts.

### Aims and research questions

This review aims to consolidate the current evidence on the application and impact of sustainability approaches in healthcare settings, including the benefits and challenges that arise through their use, and explore the sustainability outcome variables they have supported. Two research questions will be explored – (1) What sustainability approaches have demonstrated application in healthcare practice within peer-reviewed publications? (2) What is the value of using a sustainability approach? What are the documented benefits and challenges associated with the use of sustainability approaches? What impact have approaches shown on sustainability outcomes variables?

## Methods

A previous systematic review identified 62 papers that described sustainability approaches created for use in healthcare settings [27]. The sustainability approaches come in a variety of forms, including 32 frameworks, 16 models, 8 tools, 4 guidance strategies, 1 checklist and 1 process [27]. Full details on the sustainability approaches, including their constructs, scoring mechanisms and development, can be found here (10.1186/s13012-017-0707-4) [27]. This scoping review builds on these findings to explore the application and impact of these sustainability approaches in healthcare settings.

A systematic scoping review was designed to explore the available evidence on the application and impact of sustainability approaches in healthcare. To ensure overall quality and coverage of the literature, authors developed the search strategy in collaboration with a medical librarian at their academic institution. A scoping review was deemed to be the appropriate method as it allowed for examination of the “*extent, range and nature of research activity*” in the use and impact of sustainability approaches [38]. This method specifically supported authors to track the application of the 62 sustainability approaches through citation tracking and snowballing (the 62 approach articles which were tracked through the literature are listed in Additional file 1) [27].

To ensure rigorous documentation of methods, Arksey and O’Malley’s five-stage framework for scoping reviews was followed and is described below [38]. PRISMA guidelines were also used to ensure clarity of reporting [39, 40]. The PRISMA extension for Scoping Reviews Checklist can be found in Additional file 2.

### Research question(s)

The research questions were established through discussion between authors after consolidating the results from the previous systematic review, which demonstrated gaps in the current literature regarding the application and impact of sustainability approaches in healthcare settings.

### Relevant studies

Citation tracking and snowballing for each of the 62 approaches was used to determine their use in peer-reviewed publications [41]. The search was carried out between May and August 2018, with citation tracking and snowballing taking place on Web of Science and Google Scholar. This was done by putting the title and author of the sustainability approach paper into the database and selecting the ‘times cited’ or ‘cited by’ icon. Once citations were tracked for all 62 papers, we moved onto the next stage of the methodology consisting of screening the titles and abstracts.

### Data collection process and study selection

We sought peer-reviewed publications detailing the application of a sustainability approach within healthcare settings. The level of use and rationale for application were not specified but could include assessment, planning, evaluation, monitoring, prediction or testing of sustainability. Papers published in peer-reviewed journals introducing a description of the approach used as well as clear information about application were included. Papers with vague or unclear descriptions on the approach used or application were excluded. For example, papers mentioning or introducing a sustainability approach but not applying it within their studies were excluded. Papers published in languages other than English were excluded. Commentaries, research protocols, conference proceedings, editorials and perspectives were excluded. Two screening steps were undertaken. First, the selected papers went through a rapid title and abstract screening; if a sustainability approach was not mentioned in the abstract, a rapid full-text screening was conducted to confirm whether the approach was used or not within the study. Full-text screening was then conducted by two authors (LL and AL) for all eligible papers.

### Data extraction, data items and data synthesis

A data extraction table was designed to record the key components and information from the retrieved papers. This method allowed for the data to be sorted into themes and categories promoting structured analysis and interpretation [38]. Data extraction included author(s), journal, year of publication, country of study, study methodology, sustainability approach used, purpose of use, description of use, healthcare setting, timeframe, measurement, documented benefits and challenges, and reported sustainability outcome variables.

Data was independently extracted by one author (LL) on an excel spreadsheet. A second author (AL) then read through and verified the extracted information from the original full text articles.

### Quality appraisal

Although scoping reviews do not consider appraisal tools mandatory, the lack of clarity and rigour of much of the sustainability research prompted the use of a quality assessment tool within this study [17, 38, 42]. To establish study quality, the Mixed Method Appraisal Tool was used [43], which is divided into two sections. The first screening step is to ensure non-empirical studies such as reviews and theoretical papers are discounted for the analysis [44]. The screening questions are then followed by specific questions based on study methodology [43].

### Collating, summarising and reporting the results

Following the quality assessment, data extraction categories and sustainability outcomes were compared and consolidated across the included studies. Results have been summarised using descriptive statistics and narrative summaries to show the studies’ characteristics, purpose of use, the benefits and challenges of use as well as impact on sustainability outcome variables.

## Results

**Fig. 1:**
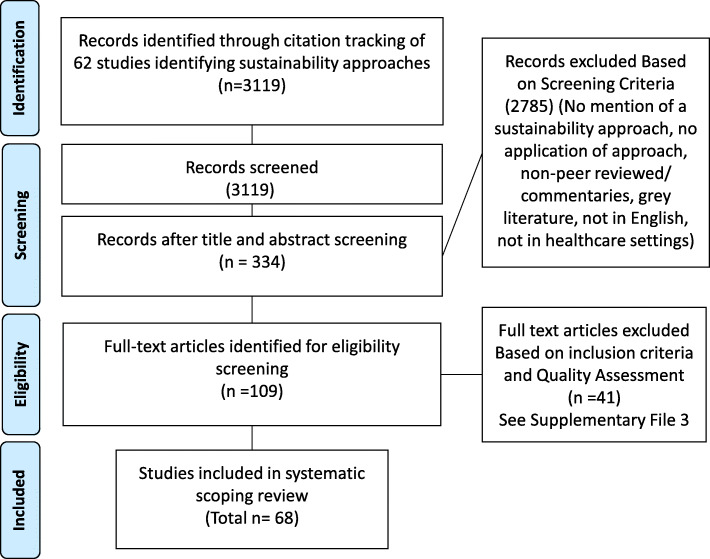
Scoping review flow diagram

The electronic citation tracking of the previously identified sustainability approaches identified 3119 publications for potential inclusion. Additional file 1 includes full information on how each of the 62 approaches were tracked through the literature. It lists the number of citations for each sustainability approach along with the number of full-text articles retrieved and included in this review. This process identified 109 eligible articles which underwent full-text screening. Following quality assessment, 41 articles were excluded based on inclusion criteria. Details on inclusions and exclusion of full text papers are included in Additional file 3. This resulted in 68 articles that were included in this review (Fig. 1). Table 1 summarises the included papers. Full data extraction (including study design, sustainability approach applied, documented benefits and challenges of use, and reported sustainability outcome variables) for the 68 articles can be found in Additional file 4.

**Table 1:** Articles included in scoping review

Author	Sustainability approach used in article	Study aim	Country	Setting
1. Ahmad M.S. & Abu Talib N.B [45].	Program Sustainability Index	*To examine the community empowerment impact on sustainability of community driven projects after decentralization in Pakistan*	Pakistan	Primary Care
2. Ahmad M.S. & Abu Talib N.B [46].	Program Sustainability Index	*To explore the relationship between community empowerment, sense of community and sustainability of community-driven projects*	Pakistan	Primary Care
3. Atkins, S. et al. [47]	Normalisation Process Model (NPM)	*To explore staff perceptions of a new TB treatment programme modelled on the antiretroviral treatment (ART) treatment programme. NPM used to highlight the experiences of staff in making the programme work in practice*	South Africa	Primary Care
4. Bamford, C. et al. [48]	Normalisation Process Theory	*To understand the barriers and facilitators to implementing nutrient- and food-based guidance for residential care homes and inform future implementation*	United Kingdom	Tertiary Care
5. Blakeman et al. [49]	Normalisation Process Theory	*To explore processes underpinning the normalisation of Chronic Kidney disease management in primary care*	United Kingdom	Primary Care
6. Blanchet K et al. [50]	The Sustainability Analysis Process (SAP)	*To use the SAP, to clarify the boundaries of systems, define sustainability, and identified sustainability indicators. To compare these definitions and use of sustainability indicators in two rehabilitation sectors*	Multiple Countries	Tertiary Care
7. Bocoum et al. [51]	Normalisation Process Model	*To describe the facilitators and barriers to the implementation of point of care testing for syphilis in antenatal care to assess the likelihood of the intervention becoming routinely incorporated in practice*	Kenya	Primary Care
8. Burau et al. [52]	Normalisation Process Theory	*To analyse the implementation of a structural health promotion intervention in community mental health organisations*	Denmark	Primary Care
9. Campbell, S. et al. [53]	Gruen’s Model of Health-Programme Sustainability	*To understand how hospitals using the Ottawa Model of Smoking Cessation (OMSC) were addressing sustainability and determine if there were critical factors or issues that should be addressed as the program expanded*	Canada	Primary Care
10. Chilundo et al. [54]	Shell’s Capacity for Sustainability Framework	*To assess the long-term success of integrated community case management (iCCM) of diarrhoea, malaria and pneumonia, by investigating programme characteristics that facilitate or impede its sustainability*	Mozambique	Primary Care
11. Coupe, N. et al. [55]	Normalisation Process Theory	*To explore to what extent Collaborative Care (CC) impacts on professional working relationships, and if CC for depression could be implemented as routine in the primary care setting*	United Kingdom	Primary Care
12. Cramm, J.M. & Nieboer, A.P [56].	Slaghuis’s Framework and Instrument for Sustainability	*To identify the predictive role of short- and long-term improvements in quality of chronic care delivery on program sustainability*	The Netherlands	Tertiary Care
13. Cramm, J.M. et al. [57]	Slaghuis’s Framework and Instrument for Sustainability	*To explore associations between partnership functioning synergy and sustainability of innovative programmes in community care*	The Netherlands	Community Care
14. Deconinck et al. [58]	Atun’s Conceptual Framework for Analysing Integration of Targeted Health Interventions into Health Systems	*To identify mechanisms promoting or hindering integration of acute malnutrition interventions into a national health system*	Niger	Secondary Care
15. Desveaux et al. [59]	Normalisation Process Theory (NPT)	*To explore how organizations respond to and interact with an accreditation process and the mechanisms through quality is influenced. NPT constructs used to categories and synthesized the themes emerging from the data*	Canada	Multiple Settings
16. Diaz del Castillo [60]	Conceptual Framework for Planning for Sustainability of Community-based Health Programs	*To identify the factors related to the sustainability and scaling up of two community-based programs offering physical activity classes in public spaces*	Colombia	Community Care
17. Dickinson et al. [61]	Normalisation Process Theory	*To explore the views and experiences of dementia care providers on the barriers and facilitators in implementing Cognitive stimulation therapy (CST) in usual care*	United Kingdom	Tertiary Care
18. Doyle, C. et al. [24]	NHS III Sustainability Model	*To describes a formative evaluation of the application of the Sustainability Model within a quality improvement Programme*	United Kingdom	Multiple settings
19. Drew, S. et al. [62]	Normalisation Process Theory	*To understand how secondary fracture prevention services can be successfully implemented to inform the implementation and integration of these services into practice*	United Kingdom	Secondary Care
20. Dugdale et al. [63]	Normalisation Process Model	*To investigate how a treatment programme for substance misuse is embedded as normal practice within Crime Reduction Initiatives*	United Kingdom	Community Care
21. Farr et al. [64]	Normalisation Process Theory	*To analyse the implementation and acceptability of the eConsult system from patient and staff perspectives using the NPT as a framework to develop coding*	Australia	Primary Care
22. Fleiszer, A. et al. [65]	Fleiszer’s Framework for the Sustainability of Healthcare Innovations	*To understand how a nursing best practice guidelines program was sustained on acute healthcare centre nursing units. To guide data collection and content analysis*	Canada	Tertiary Care
23. Ford, J.H. et al. [66]	NHS III Sustainability Model	*To measure the sustained use of Family Care Maps within Polytrauma Rehabilitation Centres*	United States	Tertiary Care
24. Fox et al. [67]	Fox’s Sustainability of Innovation Theoretical Framework	*To guide data collection, analysis and reporting*	Australia	Primary Care
25. Franx et al. [68]	Normalisation Process Theory	*To provide a better understanding of the findings the process evaluation and guide recommendations to conduct implementation projects in depression care*	The Netherlands	Primary Care
26. Furler et al. [69]	Normalisation Process Model	*To support analysis of data and develop initial coding categories*	Australia	Primary Care
27. Gask et al. [70]	Normalisation Process Model	*To inform the process of implementation of collaborative care and provide a framework to guide analysis*	United Kingdom	Primary Care
28. Gillespie et al. [71]	Normalisation Process Theory	*To provide an explanatory model to explore and evaluate the implementation of a complex intervention in the Operating Room (OR) context*	Australia	Primary Care
29. Glynn et al. [72]	Normalisation Process Theory	*To conduct a theoretically informed analysis, using NPT, of the potential barriers and levers to the implementation of a health intervention to promote physical activity in primary care*	Ireland	Primary Care
30. Godden & King [73]	Normalisation Process Model	*To explore how successful implementation of proposed new technologies could be achieved through analysis using the NPT*	United Kingdom	Primary Care
31. Green A.E. et al. [74]	Program Sustainability Index	*To examine the role of collaborations in sustaining service delivery*	United States	Primary Care
32. Herbert et al. [75]	Normalisation Process Theory	*To gain an understanding of the facilitating factors and challenges of implementing the Enhanced Recovery After Surgery (ERAS) programme using NPT to the develop the interview topic guide and to aid analysis*	United Kingdom	Tertiary Care
33. Higuchi, K.S. et al. [76]	NHS III Sustainability Model	*To examine the activities and resource implications for healthcare organisations involved in the introduction of multiple nursing guidelines, the Sustainability Model provided a framework to guide the examination of guideline implementation activities*	Canada	Multiple settings
34. Hooker, L. et al. [77]	Normalisation Process Theory	*To understand the barriers and facilitators of implementing an enhanced screening model into nurse clinical practice, NPT was used to inform the process evaluation of a pragmatic, cluster randomised controlled trial*	Australia	Primary Care
35. Ibrahim et al. [78]	Normalisation Process Theory	*To evaluate the implementation of grip strength measurement into routine clinical practice. NPT offered a framework for identifying specific factors that enabled implementation*	United Kingdom	Primary Care
36. Johnson et al. [79]	Normalisation Process Theory	*To improve the nutritional care of preterm infants by developing a complex (multifaceted) intervention. NPT used as a framework to guide implementation in order to embed the new practices into routine care.*	United Kingdom	Tertiary Care
37. Kennedy et al. [80]	Normalisation Process Theory	*To refine components of the Whole System Informing Self-management Engagement (WISE) approach. NPT provided a framework for further development of the intervention*	United Kingdom	Primary Care
28. Latter et al. [81]	Normalisation Process Theory	*To develop a pain medicines management intervention for cancer patients’ carers and evaluate feasibility and acceptability to nurses and carers. Interview guides were informed by NPT*	United Kingdom	Primary Care
39. Leon, N. et al. [82]	Normalisation Process Model	*To inform theoretical analysis of the implementation processes for Provider-initiated HIV testing and counselling (PITC) with a view to improving the optimisation of PITC scale-up in the future. NPM provided a framework to inform analysis of the implementation processes*	South Africa	Primary Care
40. Levin et al. [83]	The ARCC (Advancing Research and Clinical practice through close Collaboration) model	*To determine the preliminary effects of implementing the Advancing Research and Clinical practice through close Collaboration (ARCC) model on nurses’ EBP beliefs, EBP implementation behaviours, group cohesion, productivity, job satisfaction, and attrition/turnover rates*	United States	Primary Care
41. Lloyd, A. et al. [84]	Normalisation Process Theory	*To use NPT to examine the work that needs to be done by health professionals to inform future efforts to implement and embed shared decision making*	United Kingdom	Secondary Care
42. Mair et al. [85]	Normalisation Process Model	*To perform a process evaluation of a randomized controlled trial (RCT) of home telecare for the management of acute exacerbations of chronic obstructive pulmonary disease (COPD), using the normalization process model (NPM) as an explanatory framework*	United Kingdom	Primary Care
43. May et al. [86]	Normalisation Process Theory	*To identify factors inhibiting the implementation and integration of telecare systems for chronic disease management in the community.*	United Kingdom	Primary Care
44. Moreland-Russel et al. [87]	Program Sustainability Assessment Tool	*To identify factors that influenced the sustainability capacity of a coordinated approach to chronic disease prevention*	United States	Multiple Settings
45. Murray et al. [88]	Normalisation Process Theory	*To explore and understand the experiences of implementing e-health initiatives and assess factors which promote or inhibit the successful implementation, embedding, and integration of e-health initiatives*	United Kingdom	Multiple Settings
46. Naldemirci et al. [89]	Normalisation Process Theory	*To analyse emergent strategies adopted by healthcare professionals to overcome barriers to normalization of a specific framework of person-centred care (PCC). NPT used to analyse different challenges and strategies in a systematic fashion*	Sweden	Primary Care
47. O’Donnell and Kaner [90]	Normalisation Process Theory	*NPT used to help understand the barriers and facilitators experienced by GPs as they implemented alcohol prevention activities in routine clinical practice*	United Kingdom	Primary Care
48. O’Donnell et al. [91]	Normalisation Process Theory	*To describe and reflect on the process of designing and delivering a training programme supporting the use of theory. NPT used to shape the study approach, facilitate data collection and guide the analysis*	Multiple Countries	Multiple Settings
49. Pentecost et al. [92]	Normalisation Process Theory	*To identify a set of principles that can be built into an innovative fundamental nursing care protocol to embed the system into nursing practice. NPT used in analysis to map process data onto NPT concepts*	United Kingdom	Nursing Education
50. Redman & Barab [93]	Level of Institutionalisation Scale	*To measure the amount of routinization of diabetes education programs Maryland and Pennsylvania hospitals*	United States	Secondary Care
51. Sanders et al. [94]	Normalisation Process Theory	*NPT was used to guide analysis and explain the uptake of a new system and to examine the relevance of coherence for the implementation of innovations in organisations*	United Kingdom	Primary Care
52. Scott et al. [95]	Conceptual Framework for Sustainability of Public Health Programs	*The framework was used to inform the design of a sustainable, locally acceptable and culturally appropriate public health program using stakeholder input on factors that affect program sustainability*	Zambia	Community Care
53. Scudder et al. [96]	Program Sustainability Assessment Tool	*To examine factors impacting the sustainability of parent-child interaction therapy (PCIT) in large-scale initiatives in order to identify potential predictors of sustainment*	United States	Multiple Settings
54. Smith et al. [97]	Program Sustainability Assessment Tool	*To examine how funding influenced the delivery of fall prevention strategies and the capacity for program implementation and sustainability among the three organisations.*	United States	Multiple Settings
55. Stoll et al. [98]	Program Sustainability Assessment Tool	*To understand the factors that influenced the programs’ expected sustainability of the programs after external funding ended*	United States	Primary Care
56. Stolldorf et al. [99]	Level of Institutionalisation Scale	*To determine the level of sustainability of rapid response teams (RRTs) among a group of hospitals that participated in a state-wide collaborative to implement and sustain RRTs*	United States	Secondary Care
57. Sutton et al. [100]	Normalisation Process Theory	*To explore the utility of NPT as a methodological framework to aid exploration of Enhanced Recovery After Surgery (ERAS) programme implementation*	United Kingdom	Tertiary Care
58. Thomas, L.H. et al. [101]	Normalisation Process Theory	*To identify the organisational context for embedding the (Systematic Voiding Programme) SVP. NPT provided the theoretical framework for understanding practical issues involved in the interventions into routine practice*	United Kingdom	Secondary Care
59. Toledo Romanib et al. [102]	Level of Institutionalisation Scale	*To evaluate the sustainability of a community-based dengue control intervention over a period of 2 years after the withdrawal of external support*	Cuba	Primary Care
60. Trietsch, J. et al. [103]	Normalisation Process Theory	*To describe and analyse the challenges of embedding a quality improvement strategy to improve test-ordering behaviour of general practitioners*	The Netherlands	Primary Care
61. Underwood, M.N. et al. [104]	Leffer’s Conceptual Framework for Partnership and Sustainability	*To assess equitable partnerships between global partners to address areas of shared importance, such as equity and justice in health promotion*	Dominican Republic	Nursing Education
62. Upvall et al. [105]	Leffer’s Conceptual Framework for Partnership and Sustainability	*The purpose of this study was to revise a conceptual model of global health partnerships and collaboration integrating the perspectives of nurses from low- and middle-resource countries*	Multiple Countries	Nursing Education
63. Van Acker et al. [106]	Level of Institutionalisation Scale	*To explore the extent to which the ‘10,000 Steps’ was integrated within the culture of organizations*	Belgium	Primary Care
64. Volker et al. [107]	Normalisation Process Theory	*To determine the feasibility of translating intervention outcomes from the Model for Prevention study, into real world practice, implementation work done by stakeholders was examined using the NPT*	Australia	Primary Care
65. Walker et al. [108]	Normalisation Process Theory	*To support the development and optimisation of a Colorectal cancer RISk Prediction tool (‘CRISP’) for use in primary care. NPT used to develop interview guide and organise emerging themes*	Australia	Primary Care
66. Wallen et al. [109]	The ARCC (Advancing Research and Clinical practice through close Collaboration) model	*To guide for the development of a programme for nurses to become Evidence-based Practice mentors and champions. The ARCC model provided a framework for the development of the programme.*	United States	Secondary Care
67. Winterton and Chambers [110]	Conceptual Framework for Planning for Sustainability of Community-based Health Programs	*To explore barriers to delivering sustainable rural community programmes to increase social participation among Australian seniors*	Australia	Community Care
68. Zakumumpa et al. [111]	Level of Institutionalisation Scale	*To identify facilitators and barriers to the long-term sustainability of antiretroviral therapy (ART) programs at six health facilities*	Uganda	Primary Care

Results are presented in three sections. The first describes the application of the sustainability approaches in healthcare settings, the second discussed the reported benefits and challenges of use, and the third describes the impact of approaches on sustainability outcome variables.

### Sustainability approach application

**Fig. 2:**
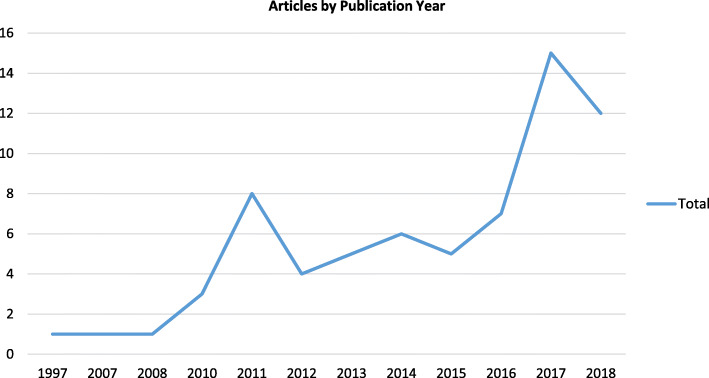
Number of articles describing the application of a sustainability approach by year

**Fig. 3:**
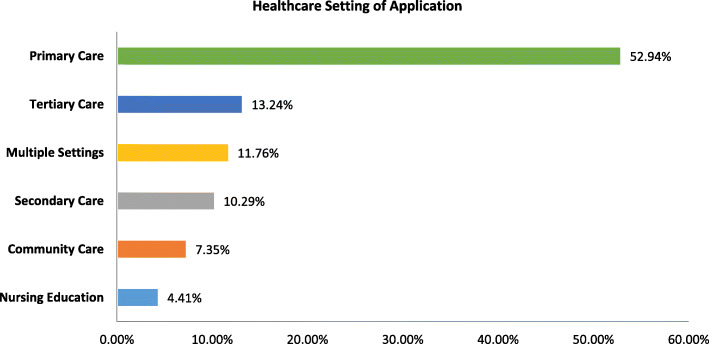
Percentage of publications by healthcare setting

Results demonstrate an increase in the use of sustainability approaches in peer-reviewed studies over time (Fig. 2). Approaches have been applied in a range of healthcare settings (Fig. 3). Most (53%, 36/68) were based in primary care settings (e.g. primary care trusts, GP surgeries, clinics, etc.). Fewer approaches were used within tertiary care (13%, 9/68) or secondary care settings (10%, 7/68). Five approaches specified that they were used in community healthcare initiatives and three approaches were used in nursing education initiatives.

The approaches have been applied in over 19 countries (Table 2). The highest proportion were conducted in the United Kingdom (34%, 23/68), followed by 15% (10/68) in the United States. Commonly, studies were undertaken within one country, but a small proportion (4%, 3/68) took place across multiple countries. While multiple study designs were found, qualitative methods were the most common, with 59% (40/68) of the studies employing methods such as interviews, focus groups and participant observation; this included four case studies [52, 54, 110, 112], two grounded theory approaches [59, 105] and one ethnography [85]. In total 28% (19/68) were mixed-method studies and 13% (9/68) were quantitative study designs using questionnaires, regression analyses or weighted scales. Most studies (79%, 54/68) explored the application of the sustainability approach, across multiple sites or organisations. For example, Campbell et al. applied Gruen’s Model of health-programme sustainability to describe the sustainability of a hospital-based smoking cessation program across six Canadian hospitals [53]. Finally, 21% (14/68) were used within single sites (e.g. intensive care units, care homes, GP clinics, etc.).

**Table 2:** Number of publications by country location

Country	Number of publications	Country	Number of publications
United Kingdom	23	Mozambique	1
United States	10	Kenya	1
Australia	8	Belgium	1
Canada	4	Uganda	1
The Netherlands	4	Niger	1
Pakistan	2	Denmark	1
South Africa	2	Ireland	1
Colombia	1	Dominican Republic	1
Zambia	1	Sweden	1
Cuba	1	Multiple Countries	3

#### Sustainability approaches applied in reviewed studies

Within the 68 reviewed articles, 17 different sustainability approaches were applied. Table 3 summarises the sustainability approaches demonstrating use within the reviewed publications along with the purpose and constructs included within each approach. The most commonly applied approach was the Normalisation Process Theory (NPT), which was used to in 30 articles. The Normalisation Process Model, Level of Institutionalisation (LoIn) Scale, Program Sustainability Assessment Tool, Program Sustainability Index and the NHS III Sustainability Model were all used within 3 or more studies. The remaining 11 approaches were used in 2 or fewer articles.

**Table 3:** Sustainability approaches demonstrating use in practice

Sustainability approach	Approach purpose	Sustainability constructs	Number of Articles
1. Normalisation Process Theory [113]	To explore the social organisation of the work (implementation), of making practices routine elements of everyday life (embedding) and of sustaining embedded practices in their social contexts (integration)	Coherence (or sense-making), cognitive participation (or engagement), collective action (work done to enable the intervention to happen), and reflexive monitoring (formal and informal appraisal of the benefits and costs of the intervention)	30
2. Normalisation Process Model [114]	To assist in explaining the processes by which complex interventions become routinely embedded in healthcare practice	Interactional workability, relational integration, skill-set workability, contextual integration	8
3. Level of Institutionalisation Scale [115]	To measure the extent of programme integration into an organisation	Production routine, production niche saturation, maintenance routine, maintenance niche saturation, supportive routine, supportive niche saturation, managerial routine, managerial niche saturation	5
4. Program Sustainability Assessment Tool [116]	To assess and plan for sustainability risks and develop an action plan	Political support, funding stability, partnerships, organisational capacity, programme evaluation, programme adaptation, communications, strategic planning	4
5. Program Sustainability Index [23]	To evaluate community-based programme sustainability	Leadership competence, effective collaboration, understanding the community, demonstrating programme results, strategic funding, staff involvement, integration, programme responsivity	3
6. NHS III Sustainability Model [20]	To predict the likelihood of sustainability and guide teams to things they could do to increase the chances that the change for improvement will be sustained	Staff involvement and training, staff attitudes towards sustaining the change, senior leadership engagement, clinical leadership, fit with the organisation’s strategic aims and culture, infrastructure for sustainability, benefits beyond helping patient, credibility of the benefits, adaptability of improved process, effectiveness of the system to monitor progress	3
7. Slaghuis’s Framework and Instrument for Sustainability [117]	To analyse sustainability of actual changed work practices and evaluate improvement projects	Routinisation I (principle forming practice), Routinisation II (variations in practice), routinisation III (feedback on performance), institutionalisation of skills, institutionalisation of documentation materials, institutionalisation of practical materials, institutionalisation of team reflection	2
8. The ARCC (Advancing Research and Clinical practice through close Collaboration) Model [118]	To provide healthcare systems with a conceptual framework to guide system-wide implementation and sustainability of evidence-based practice (EBP) for the purpose of improving quality of care and patient outcomes	Culture, organisational readiness, Philosophy of EBP Presence of EBP mentors and champions, administrative support, EBP knowledge and skills, EBP value, ability to implement the EBP process	2
9. Conceptual Framework for Planning for Sustainability of Community-based Health Programs [1]	To conceptualise and measure sustainability with tentative guidelines to facilitate sustainability in community programmes	Project negotiation process, project effectiveness, project duration, project financing, project type, training, institutional strength, integration with existing programs/services, programme champion/leadership, socioeconomic and political considerations, community participation	2
10. Leffer’s Conceptual Framework for Partnership and Sustainability [119]	To offer guidance and a framework for partnership and sustainability for nurses who participate in global efforts	Design and implementation, community assessment, organisational setting, resources, broader host community, social and political climate, community participation, Processes: leadership champion, Outcomes, project ownership	2
11. Atun’s Conceptual Framework for Analysing Integration of Targeted Health Interventions into Health Systems [120]	To analyse and map the nature and extent of integration in different settings, along with the factors that influence the integration process	Nature of the problem, the intervention, the adoption system, health system characteristics, context	1
12. Shell’s Capacity for Sustainability Framework [121]	To provide a framework on sustainability capacity, identifying organisational and contextual characteristics necessary for successfully sustaining programmes over time	Political support, funding stability, partnerships, organisational capacity, programme evaluation, programme adaptation, communications, public health impacts and strategic planning	1
13. Fox’s Sustainability of Innovation Theoretical Framework [122]	To guide research, determine variables, influence data analysis	Political factors (policy alignment, link with visions and goals, champion involvement, staff involvement), organisational (communications, adaptation of the innovation, networking opportunities), financial (funding sources, budgetary planning, evaluation strategies), workforce (staff recruitment, education and training, perception of need for innovation, perception of quality and safety), innovation (support for innovation, barriers, quality and safety)	1
14. Conceptual Framework for Sustainability of Public Health Programs [17]	To guide the sustainability research agenda and enable accumulation of findings about sustainability.	intervention with evidence for effectiveness, organisational capacity, prior relationships and partnerships, intervention characteristics, organisational support, environmental support, financial resources	1
15. Gruen’s Model of Health-Programme Sustainability [22]	To provide a model of health-programme sustainability based on context and resource availability	Health concerns, programme elements, drivers of the programme, context, resource availability	1
16. The Sustainability Analysis Process [123]	To conceptualise and measure the sustainability of health systems in low-income countries and fragile states	Sustainability indicators and characterisation are developed by users and based on the the local context and setting	1
17. Fleiszer’s Framework for the Sustainability of Healthcare Innovations [124]	To guide data collection and content analysis	Culture, interprofessional collaboration, financial resources, external pressure, extra-organisational partnerships, relevance of the programme, nature of the programme, reflection strategy, co-directorship of the programme, commitment of leaders, complementarity of leadership actions	1
**Total**			**68**

#### Purpose of application

**Fig. 4:**
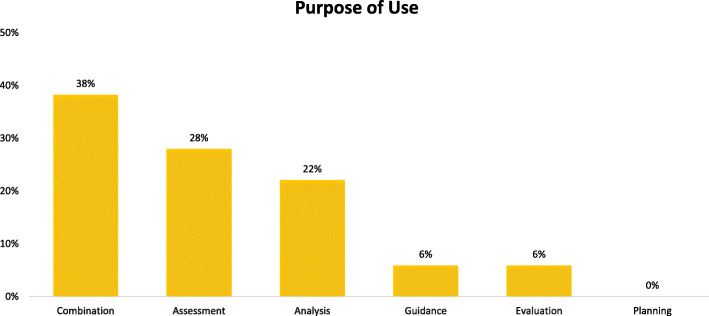
Purpose of use described in articles

The purpose of using a sustainability approach fell into five categories – analysis (to examine and interpret data in relation to sustainability), evaluation (to appraise sustainability of an existing programme), guidance (to direct study design, data collection, discussion, etc.), assessment (to measure constructs for sustainability) and planning (to design a programme to achieve sustainability). Twenty-six (38%) articles used a sustainability approach for a combination of these purposes such as planning and evaluation or guidance and analysis (Fig. 4). For example, Johnson et al. used NPT to both guide and assess sustainability of an intervention to improve the nutritional care of preterm infants [79]. Nineteen of the articles (28%) used a sustainability approach for assessment. For example, Cramm et al. used Slaghuis’s Framework and Instrument for Sustainability to assess connections between partnership functioning and sustainability of innovative programmes in community care [57]. Fifteen studies (22%) used an approach to inform data analysis for the study. This was demonstrated in Toledo Romanib et al.’s study where the LoIn scale was used to guide content analysis [102]. No study used a sustainability approach exclusively for planning for sustainability, but planning was reported in combination with other purposes.

### Benefits and challenges

Details on and documentation of the application of the sustainability approaches varied across the studies. While 65% (44/68) of articles identified benefits associated with the use of approaches, only 34% (23/68) reported challenges. Table 4 reports findings on the benefits and challenges for the individual approaches. This is followed by discussion of six common themes related to the benefits and challenges found across sustainability approaches.

**Table 4:** Benefits and challenges reported for individual approaches

Sustainability approach	Benefits	Challenges
1. Normalisation process theory	• Aided users to expose the ‘hidden work’ that needs to occur to create health-promoting systems • Created understanding of the barriers to implementation and identified potential strategies to address barriers • Provided framework to organise findings • Facilitated analysis of implementation from multiple perspectives and understanding of experiences of healthcare workers at the individual and organisational level • Drawing planners’ attention to potential problems to address during implementation	• Further development needed to link constructs to specific behaviour-change techniques • Overlap and difficulty of discerning the difference between the constructs • Based on perceptions of individual users; therefore, risk of bias and leaving some contextual factors beyond the scope of knowledge
2. Normalisation process model	• Allowed the identification of barriers and facilitators impacting the programme • Provided framework to organise findings • Facilitated a deeper and more dynamic analysis	• Difficult to assign to a single category to the data as categories overlap
3. Level of institutionalisation scale	• Allowed an aspect of continuous evaluation by measuring whether intervention is becoming institutionalised • Provided insight on implementation problems in routine settings • Enabled exploration of programme sustainability at different levels of care • Identified risks and barriers to sustainability	• More work is needed to test with larger samples and different health promotion programmes • Does not measure the processes leading to institutionalisation, only whether structural components are present or not present • Wording and response options may need to be modified to fit with specific contexts
4. The advancing research and clinical practice through close collaboration model	• Improved outcomes of believing in the value of evidence-based practice and increased reported use of evidence-based practice implementation behaviours • Led to positive effects on nurses’ perceptions of organisational culture and readiness, beliefs and implementation, job satisfaction, group cohesion	• The model should include a cost component and patient outcomes to evaluate potential savings
5. Program sustainability assessment tool	• Provided an overview of sustainability strengths and weaknesses • Determined programme elements intended to sustain for the long term • Enabled team to demonstrate accomplishments, tell their story, and build connections • Facilitated the development of a vision and mission for the programme	• The brevity and simplicity of the tool may not capture the nuances of the setting and situation
6. Program sustainability index	• Provided evidence of the supporting role of effective collaborations in sustainment across different service systems • Provided a form of measurement	• Scales may require adaptation for use
7. NHS III sustainability model	• Created an understanding of determinants of sustainability • Found to be relevant for examining implementation processes across a range of clinical settings	• Aspects of the model’s design should be considered to include more user-friendly design • Needs greater emphasis on the political and economic environment as well as patient and public engagement
8. Slaghuis’s framework and instrument for sustainability	• Highlighted that strong relationships connect partnership functioning, synergy and the sustainability of innovative programmes in community care • Identified short and long-term improvements in quality of chronic care delivery predicted programme sustainability	• Lack of relevance of specific subscales
9. Shell’s capacity for sustainability framework	• Highlighted key strengths and weaknesses as well as levers within programmes	• Some domains need further conceptual refinement • Hard to categorise domains as entirely positive or negative due to the many nuances involved
10. Leffer’s conceptual framework for partnership and sustainability	• Provided the structure for deeper understanding of distinctive views regarding the engagement processes and partner factors for effective collaboration • Model constructs offered a platform to engage in dialogue with partners to gain context-specific insights • Useful in guiding study to examine global health partnerships	• Model did not explore nurse partner factors, resources or sustainability; therefore, applicability of the model with other host partners, professions and contexts needs to be investigated • Not generalisable to other countries outside of the United States
11. Gruen’s model of health-programme sustainability	• Provided greater insight into the sustainability of interventions • Provided insight into issues affecting programme sustainability and may foster development of a sustainability plan	• Not stated
12. Fleiszer’s framework for the sustainability of healthcare innovations	• Aided in the identification of characteristics of programme sustainability	• May benefit from further investigation to examine the long-term sustainability and discontinuation of different kinds of innovations in diverse settings
13. The sustainability analysis process	• Supported participants to clarify the boundaries of their systems, define sustainability and identify sustainability indicators	• Not stated
14. Conceptual framework for planning for sustainability of community-based health programs	• Considered useful for analysis • Provided an understanding of how programme sustainability is impacted by different components in and out of the community	• Framework could not address cultural specificity
15. Atun’s conceptual framework for analysing integration	• Provided a systems lens for increasing integration and how this can help sustain effective interventions	• Not stated
16. Practical, robust implementation and sustainability model	• Provided valuable data that helped develop a detailed implementation plan and facilitated the implementation process	• Not stated
17. Conceptual framework for sustainability of public health programs	• Useful in explaining sustainability	• Not able to explain all financial sustainability strategies

#### Benefits of use

For the 65% (44/68) of articles describing benefits of use, three main benefits were found, namely (1) improved conceptualisation of sustainability, sustainability constructs and their impact on systems, (2) improved ability to organise, analyse and interpret sustainably data, and (3) increased focus on areas for improvement.

**Improved conceptualisation of sustainability, sustainability constructs and their impact on systems: 37% (25/68)**

The most common benefit reported from the use of sustainability approaches was the improved understanding of sustainability and sustainability constructs. Twenty-five articles stated they were able to improve conceptualisation of sustainability of interventions [47, 48, 50, 53, 54, 58, 61, 71–73, 75, 78–80, 86, 88, 92, 95, 96, 100, 101, 103, 104, 110]. For example, Deconinck et al. applied Atun’s Conceptual Framework for Analysing Integration of Targeted Health Interventions, and found that the approach provided a systems lens that increased understanding of pathways for integration to help sustain coverage of effective interventions [58, 120]. Some articles also explicitly reported gaining a better understanding of barriers and facilitators to sustainability of their initiatives. The Program Sustainability Assessment Tool was used to help predict an initiatives’ integration in Scudder et al.’s study which found barriers in financial support and implementation funding [96, 116].

**Improved ability to organise, analyse and interpret sustainably data: 26% (18/68)**

Many of the approaches were used to guide study design and analysis. In this process, authors noted that the approaches aided analysis and interpretation of data [52, 55, 60, 63, 64, 68, 70, 76, 77, 81, 89, 94, 98, 105, 107, 108, 111]. For example, within Sanders et al.’s article, the NPT was used to organise their semi-structured interview data [94]. Authors noted that the NPT not only aided in the design of the interview but also guided thematic analysis on improving uptake of the new system for general practitioners. Similarly, Zakumumpa et al. found that the LoIn Scale aided them to analyse antiretroviral therapy institutionalisation scores across multiple cases [111].

**Increased focus on areas for improvement: 9% (6/68)**

Finally, the use of sustainability approaches was beneficial to improve focus and highlight areas needing further work [56, 62, 66, 69, 82, 85]. This was highlighted within Ford et al.’s study using the NHS III Sustainability Model. This study found that the model provided a measurement indicating where a component in the organisation needs improvement for sustainability [66]. Similarly, Blanchet et al. noted that the Sustainability Analysis Process aided them to better understand the need for participation of major stakeholders to promote sustainability within the rehabilitation sector [50].

#### Challenges of use

Key challenges were reported in 23 articles. These included (1) issues with approach constructs, (2) need for adaptation or improvement and (3) difficulty in application.

**Issues with approach constructs: 16% (11/68)**

The most common challenge associated with the use of sustainability approaches was associated with approach constructs. Issues such as applicability, relevance, overlap or missing constructs were encountered across a number of studies [47, 62, 70, 71, 82, 95, 96, 100, 104, 107, 109]. For example, users found difficulty in discerning the differences between the constructs within the NPT, which were often reported to overlap in content and concept. Both Drew et al. and Gillespie et al. found that the overlapping nature of the four constructs within the NPT meant the data could be coded into more than one construct [62, 71]. Another noted issue was the inability of constructs within approaches to cover all the relevant areas needed for studies [82, 104, 109]. This was identified within Underwood et al.’s study, which found that Leffer’s Conceptual Framework for Partnership and Sustainability did not adequately explore resources or partner factors [104].

**Need for adaptation and improvement: 10% (7/68)**

Seven studies noted that the approaches were a challenge to be used as originally designed and suggested areas for improvement [24, 46, 48, 68, 79, 85, 93]. For example, the Program Sustainability Index was used by Ahmad and Abu Talib to assess community capacity. During the application, authors stated that the index had to be adapted to suit the study needs [46]. A number of studies also recommended improvements for approaches to work more effectively. Redman and Barab suggested the LoIn scale required further testing with larger samples and different health programmes to improve its applicability [93]. Bamford et al. suggested that the NPT may require further development to explore specific behaviour change techniques to increase the practical value of the theory [48].

**Difficulty in application: 9% (6/68)**

It was noted within six papers that authors encountered some difficulty in applying the approaches [24, 52, 77, 91, 97, 110]. In Winterton and Chambers’ study, they found that Shediac-Rizkallah and Bone’s conceptual framework was difficult to apply to initiatives in certain ethnic populations [1, 110]. Doyle et al. reported similar findings with the NHS III Sustainability Model. In this study, users reported concerns over aspects of the model design and only 12 of the 19 teams found it acceptable for routine use [24, 125].

### Examining impact on sustainability outcome variables

To understand potential links between the use of a sustainability approach and enhanced reporting of or impact on sustainability, six sustainability outcome variables were investigated. The majority (79%, 54/68) did not report sustainability outcome variables in their studies. Low reporting was attributed to how the approaches were used in practice as many articles applied approaches to aid conceptualisation of sustainability constructs; therefore, sustainability outcomes were not explicitly assessed. Only 21% (14/68) reported one or more sustainability outcome variable. Table 5 summarises the articles that reported sustainability outcomes. Below each outcome variable is discussed with specific examples of how each was reported within the studies.

**Table 5:** Articles reporting sustainability outcome variables

	Sustainability outcome variables reported						Additional sustainability outcomes reported		
Author and sustainability approach used	Benefits for patients, staff and stakeholders continue	Initiative activities or components of the intervention continue	Maintenance of relationships, partnerships or networks	Maintenance of new procedures, and policies	Attention and awareness of the problem or issue is continued or increased	Replication, roll-out or scale-up of the initiative	Capacity built within staff, stakeholders and communities continues	Adaptation in response to new evidence or contextual influences	Gaining further funds to continue the initiative and maintain improvements
Blanchet et al. [50]			✓	✓				✓	✓
Diaz del Castillo et al. [60]			✓		✓	✓	✓	✓	✓
Ford et al. [66]	✓			✓			✓		
Higuchi et al. [76]	✓	✓					✓		
Ibrahim et al. [78]		✓							
Johnson et al. [79]	✓			✓					
Leon et al. [82]		✓		✓					
Lloyd et al. [84]	✓	✓							
Moreland-Russel et al. [87]			✓					✓	
Redman & Barab [93]		✓		✓					
Scudder et al. [96]		✓	✓		✓	✓		✓	
Stolldorf et al. [99]		✓		✓			✓		✓
Toledo Romanib et al. [102]	✓	✓	✓		✓		✓		✓
Van Acker et al. [106]		✓							
Total number of studies reporting outcome variable	6	9	5	7	3	2	5	5	4

### Continued programme activities or components of the original intervention (9/14)

The most common outcome reported across the studies was continuation of programme activities or components, with nine articles exploring the continuation of initiative activities [76, 78, 82, 84, 93, 96, 99, 102, 106]. ‘Continuation of activities’ was demonstrated through multiple measures, including continuation of services (e.g. continued delivery of the HIV testing service [82]), tool use (e.g. continued use of a shared decision-making tool [84]) and continued delivery educational programmes for staff and patients (e.g. diabetes education programmes [93]). Three of these detailed partial continuation of activities [78, 99, 106]. For example, Redman et al.’s study using the LoIn scale found ‘low to moderate levels’ of routinisation were achieved, which suggested that the programmes had embedded into the host organisations [93]. Similarly, Ibrahim et al. discussed partial continuation of a grip strength measurement programme across five acute medical wards with weekly coverage of the programme ranging between 65% and 100% in one site and between 0% and 93% in another [78].

### Maintenance of new organisational practices, procedures and policies that were started during programme implementation (7/14)

Maintenance of new practices and policies was highlighted in six articles [50, 66, 79, 82, 93, 99, 102]. This variable was demonstrated through intervention integration within clinical guidelines [79, 82], electronic records [66], through ongoing discussion at team meetings [66] and monitoring/evaluating activities [99]. For example, Toledo Romani et al.’s study using the LoIn scale found that elements of the intervention (community working groups and provincial/municipal coordination groups) had lost their separate identity and had become part of the programme’s regular procedures [102]. Blanchet et al. followed two rehabilitation sectors using the Sustainability Analysis Process for 2 years. At follow-up, they noted that, although there were differences in the structure of social networks, the programme policies continued to be used [50].

### Whether benefits or outcomes for consumers, clients or patients are continued (6/14)

Six articles reported continued benefits to patients [66, 76, 79, 84, 102, 106]. Detail on these benefits was variable across the studies. Some reported specific measures of continuation of benefits such as continued behaviour change or continuation of improved patient outcomes [102]. For example, Toledo Romani et al. used the LoIn scale and demonstrated maintenance of behavioural changes related to a dengue control intervention [102]. Johnson et al. used the NPT to study guideline compliance and found that the intervention resulted in maintenance of improvements in health outcomes for infants, specifically protein intake and weight gain [79]. Others reported continuation of benefits in terms of continued support of patients and families (e.g. a shared decision-making tool helping patients to choose treatment options [84] or family care maps supporting families and patients on the continuum of rehabilitation process [66]). Higuchi et al. used the NHS Sustainability Model and reported vague continuation of benefits. They described how sustained organisational perceptions of benefits existed 5 years after their initial initiative to introduce multiple nursing guidelines [76]. A number of studies also described the benefits to other stakeholders affected by the programme, such as their staff, communities and carers [65, 66, 79].

### Maintenance of community-level partnerships or coalitions developed during the funded programme (5/14)

Five articles reported the maintenance of partnerships and described how it was beneficial or challenging for the continuation of the programme [50, 60, 87, 102]. This variable included the active continuation of stakeholder networks, champion engagement, leadership groups, steering committees and building further collaborative relationships. The maintenance of partnerships was highlighted in Diaz del Castillo et al.’s study where champions were a core part of the improvement delivery. The programme was grounded in the work of champions, who would continuously advocate, garner further resources, recommend strategies and implement activities [60]. Scudder et al. also discussed the importance of partnerships in sustaining a parent-child interaction therapy programme. They noted that the leadership groups and steering committees developed during the maintenance period were key sustainability outputs from the programme. Blanchet et al. found similar importance of developing strong partnerships and networks but noted that these relationships can also be a challenge to sustain. Within this study, one site documented the breakdown of their stakeholder network, which impacted their ability to continue to collect sustainability indicators [50].

### Sustained attention to the issue or problem (3/14)

Three studies highlighted that attention to the issues or problem was sustained [60, 102]. This was demonstrated through the use of national campaigns and goals [60], mass media and TV campaigns [102], and community meetings [102]. Diaz del Castillo reported that their programme was able to sustain attention through involvement in national campaigns where their ‘Healthy Habits and Lifestyles Program’ was included in the Ten-Year National goals [60]. Scudder et al.’s study also reported maintaining attention to their programme through a number of strategies, including ‘word of mouth’ and TV campaigns.

### Programme diffusion and replication in other sites (2/14)

Two studies reported programme diffusion or replication [60]. Scudder et al.’s study demonstrated diffusion of their programme across the United States. The authors noted that the original programme was replicated but the strategies for integration were adapted to meet the needs of other systems, organisations or populations involved. Diaz Del Castillo et al. reported that their programme grew from 12 locations in 1996 and to 31 locations in 2005. They stated, “*programs have lasted more than 10 years, scaled-up locally/nationally, and exist in similar forms elsewhere*” [60]*.*

#### Additional sustainability outcome variables

Throughout data extraction, it was noted that several articles discussed and reported sustainability outcome variables that did not fit into the six variables explored [17]. Three emerging outcomes reported across various studies are discussed below.

**Capacity built (5/14)**

This outcome was discussed within five studies [60, 66, 76, 99, 102] in relation to how the skills and capabilities developed throughout an initiative could continue to be utilised and developed after the end of initiative funding. This variable refers to the development of strengths, skills and abilities of staff, stakeholders and communities to maintain new organisational practices that were started, through their involvement with an improvement initiative [126]. This was demonstrated through integration of intervention into training programmes and orientations [66, 99, 102], and attending knowledge exchange events [76]. Diaz del Castillo et al.’s study specifically commented on the continuation of capacity built and skills gained in the initiative [60]. Authors reported that programmes had expanded and been maintained due to the quality of the instructors, which was seen to impact both people’s continued attendance and perceived benefits. The programme maintained training for staff with a ‘Teachers’ Academy’ created to select and train instructors for the programme [60].

**Innovation and Adaptation (5/14)**

This outcome relates to the fidelity and flexibility of initiatives and the ability to respond and adapt to challenges and changing needs. While most studies that were reviewed did not describe adaptations or examine their impact on health-related outcomes, this variable was discussed in select studies [50, 60, 87, 106]. Specifically, adaptations to system characteristics [50], funding changes [60], internal culture [87], existing needs and challenges and products (e.g. changing campaign images [106]) were noted. For example, Scudder et al.’s study found that, while initiatives were actively sustaining, no clear strategies were linked to their success but rather, “*the common theme across initiatives was the importance of being responsive to existing needs and challenges*”. Diaz del Castillo also found that, for both programmes in their study, there was “*a need and willingness to be flexible*” [60]. Flexibility, in this case, meant adapting programmes to people’s preferences, funding changes and diverse scenarios/conditions [60]. Blanchet et al. also noted how adaptation and resilience played a part in the sustainability of improvements with two rehabilitation sectors. They noted that “*the structure of the rehabilitation system characteristics evolved over time and helped understand the changing nature of relationships between actors and their capacity to work as a system. This aided in the creation of consensus on a common vision of sustainability*” [50]. To facilitate a greater understanding through future research, some clarity regarding adaptation and fidelity is necessary [3].

**Ability to garner further funding (4/14)**

An initiatives’ ability to garner further funds to continue the initiative and maintain gains was also reported as a sustainability outcome across several studies [50, 60, 99, 102]. Toledo Romani et al. reported how funds for the activities of the community dengue intervention were included in the annual budget of the wider dengue control programme as an action towards sustainability [102]. This was also highlighted in the Diaz Del Castillo study where they described how 19 of 32 departments had co-funded programmes and six programmes operated using their own resources. They highlighted that “*the creation of a specific PA (Physical Activity) unit in 2011 led to a dedicated budget, resources, goals and a capacity to translate policy into action*” [60]. Blanchet et al. also identified gaining further resources as a mechanism indicating sustainability and noted that “*financial support by an international organization facilitated advancement toward self-identified sustainability goals*” [50].

## Discussion

While early implementation research was criticised for a lack of attention to the theoretical underpinnings, in the last two decades, numerous theories, models and frameworks have been produced [19]. Due to the vast number of approaches available, choosing the most appropriate one is increasingly difficult [30, 31]. To understand the value of models and frameworks, we require supporting evidence to warrant their use [32]. This review provides healthcare professionals and researchers with the current evidence on sustainability approaches, including the benefits and challenges that arise through their use and the outcomes they have supported.

This review tracked sustainability approaches in the literature and found 68 articles detailing their use in peer-reviewed publications. It found that, while many sustainability approaches are available, few have demonstrated their practical application or benefits, with only 17 of the 62 available approaches showing use in peer-reviewed publications [27]. Sustainability approaches were used to assess, evaluate, analyse and guide sustainability measurement and research. Benefits of use include improved understanding of the barriers and risks to sustainability, improved conceptualisation of sustainability and its constructs and improved ability to organise, analyse and interpret sustainably data. The literature has also documented challenges, including issues with approach constructs, difficulty in application and need for improvement in design [4, 54, 62, 82, 127]. These results support previous findings reporting that tools, frameworks and models may improve team knowledge and enhance action but they can also be complicated to use, hinder teamwork and take substantial amounts of time [21, 46, 48, 54, 62, 82, 127–132]. The findings presented in this study, along with these previous reports, demonstrate the need for practitioners and researchers to have clear and realistic expectations when using frameworks, models or tools for sustainability. It is important to not only understand the potential benefits but also the challenges to realistically consider application and weigh potential issues with expected impact [3, 35, 36].

### Implications for future measurement

This study has shown that, while there is increasing interest in the use of sustainability approaches, there is little reported evidence of their impact on sustainability outcome variables [21, 26, 36, 116, 133, 134]. Although most sustainability approaches are intended to support or influence long-term success, only 21% (14/68) of studies reported any information on sustainability outcomes. This is similar to the results reported by Stirman et al. in their systematic review, which found only 22% of studies reporting information about sustained outcomes [3].

The low reporting of sustainability outcome variables is often attributed to poorly defined concepts of what sustainability means within individual studies [36]. This issue is compounded by the frequent reporting of sustainability as a dichotomous measure [135]. For example, in Leon et al.’s, study they concluded that their intervention had become sustained and was “*embedded in practice (normalised) during a two-year period*” but information on what was embedded, what specific activities were continued, and what other variables were sustained were not discussed [82]. These findings highlight that, although the use of multiple outcome variables to report sustainability outcomes is recommended, it is not common practice [17].

Future research should endeavour to clearly describe and outline expected sustainability outcomes and consider the use of multiple outcome variables to capture comprehensive sustainability narratives [17]. Reporting dichotomous or unclear outcomes contributes to underreporting of sustainability failures and successes, making it increasingly difficult to understand the true scale of the problem, compare outcomes across studies or provide generalised learning on sustainability measurement.

While this study examined six sustainability outcome variables, three additional variables were discussed in our results, namely Capacity built, Innovation and adaptation, and Garnering funding. Although not part of Scheirer and Dearing’s original six variables, these outcomes have been suggested elsewhere as being potential variables that are sustained following healthcare initiatives [1, 21, 22, 116, 121, 136–138]. These additional outcomes may lend a new understanding of how sustainability outcomes can be viewed, assessed and reported in practice.

The role of continued innovation and adaptation in assessing sustainability is of particular interest to those adopting a ‘process’ view of sustainability. Much of the previous research considered deviations from intervention protocols to be implementation failure but, due to growing recognition that institutionalisation of activities may impede organisations from adopting more effective practices, there is now greater acceptance of the role of adaptation and capacity-building in sustainability [3, 4, 22, 36, 139]. Unfortunately, few studies explore what adaptations are made to interventions, why such changes are necessary, and how decisions to change intervention components are made [3, 17, 140]. Future research should prioritise studying this process to provide insight into how adaptations may impact outcomes and sustained services [3, 141, 142].

### Implications for practitioners and researchers

It is recommended “*that researchers specify the unit of data collection and analysis for both sustainability determinants and outcomes*” [36]. This review seeks to support this further by incorporating emerging findings to guide measurement and study of sustainability in healthcare settings by providing an updated list of sustainability outcome variables (Table 6). It is hoped that having a clear list of potential outcomes to assess will support practitioners and researchers to clearly define the sustainability outcome variables of interest and consider how evidence for each outcome may be collected and analysed. It also aims to support the consideration of a wide breadth of sustainability outcomes to discourage unclear, over simplistic or dichotomous reporting of outcomes. While all variables may not apply to all settings, this list can serve as a baseline for sustainability outcome planning and measurement. To understand its potential value in achieving these aims, this list requires further testing and validation within future studies. Specifically, there is a need for future work to provide insight into how data on each of these variables can be collected and analysed.

**Table 6:** Updated list of sustainability outcomes variables

Updated sustainability outcome variables		
Sustainability outcome Variable	Description	Example
I. Benefits	*Benefits for patients, staff and stakeholders continue*	e.g. continued improvements in health outcomes for infants in protein intake and weight gain [79]
II. Activities	*Initiative activities or components of the original intervention continue*	e.g. continuation of the HIV testing service delivery [82]
III. Relationships, partnerships and networks	*Maintenance of relationships, partnerships or networks that were developed during the funded programme*	e.g. maintenance of relationships with champion groups to continuously advocate for and implement activities [60]
IV. Procedures and policies	*Maintenance of new organisational procedures, and policies that were started during program implementation*	e.g. workflow integration achieved through ongoing communication and documentation in electronic records [66]
V. Attention and awareness	*Attention and awareness of the problem or issue is continued or increased*	e.g. using educative messages through mass media and community meetings [102]
VI. Spread	*Replication, roll-out or scale up of the initiative*	e.g. programme is scaled-up locally/nationally and exist in similar forms elsewhere [60]
VII. Skills and capabilities	*Capacity built (skills and capabilities) within staff, stakeholders and communities developed throughout an initiative continue to be utilised*	e.g. intervention is incorporated into staff orientation [66]
VIII. Innovation and adaptation	*Adaptation in response to new or changing populations, evidence, policies, or other contextual influences*	e.g. replacing intervention posters in public places or changing intervention images [106]
IX. Garnering funding	*Gaining further funds to continue the initiative and maintain improvements*	e.g. funds for the intervention activities are included in the annual budget for illness prevention strategies [102]

Given this expanded list of outcome variables, planning for their collection and analysis may be a challenge. The most appropriate methods for collecting evidence related to each of these variables has not been determined but it is increasingly recognised that sustainability research should draw on mixed methods [3, 13, 17, 143]. A mixed methods approach supports both quantitative methods key to measuring ongoing performance of initiatives (e.g. continuation of benefits or activities) as well as the qualitative methods to provide the necessary ‘texture and detail’ to describe intervention sustainability (e.g. continued partnerships or training) [143].

#### Future research

While this review presented the available evidence on the application and impact of sustainability approaches in healthcare, it also highlighted the remaining gaps in the literature. First, there is a need for future research to more rigorously report and explicitly describe how frameworks, tools and models inform development, organisation and implementation of research [7, 19, 27, 34]. This evidence will help to support improved sustainability approach development and improve the reliability of sustainability studies [3, 19, 35, 36]. Further work is also needed to build an understanding of under-use and misuse of frameworks, models and tools [29]. This study found that sustainability approaches were largely used for analysis and conceptualisation purposes to aid in interpretation and organisation of data. This does not reduce the value of these studies but highlights the challenge of understanding the impact of these approaches if they are not used as fully intended. For example, the aim of the NPT is to help understand how complex interventions can embed change and become ‘normalised’ in practice [113]. Although this approach has the potential to explain how complex interventions become routinely embedded, the evidence shows that it is often used to assess implementation processes only. Future studies may benefit from reporting on all aspects of the approach, including embedding and integration. There is also a need to empirically test the updated sustainability outcomes list to assess if it clarifies what continued, discontinued or changed for interventions and if it provides better insight into sustaining initiatives. This will be critical in understanding the process and potential difficulty of studying these diverse sustainability outcomes in practice.

#### Strengths and limitations

This is the first review to examine the application and impact of sustainability approaches in healthcare and the first review attempting to understand the impact of sustainability approaches on sustainability outcomes.

There are a number of limitations associated with this systematic scoping review that are must be acknowledged. The first limitation is the potential for publication bias which may have manifested in a number of ways. First, the search strategy may have missed published articles if they had not referenced the use of an approach within their study. Second, this review only drew on the 62 approaches identified in a previous systematic review, which meant that approaches published after 2018 were not included in this work. This was largely a pragmatic decision as we believed the existing 62 approaches would provide sufficient evidence of the scope of application and impact of approaches. It was also believed that newer approaches would not have had sufficient time to build evidence of their impact within this short timeframe. Therefore, future work should continue to assess and collect information on emerging approaches and the evidence of their application. Finally, the review did not search the grey literature, which means that findings may be more representative of academic and research application and analysis.

While there is value in consolidating insight into the perceived experience of implementing such approaches, another limitation to this work is the reliance on reported benefits and challenges. Many papers did not discuss benefits or challenges and those that did may not have been comprehensive in their descriptions as this was not the primary aim of their work. This may have resulted in over or under reporting of benefits and challenges.

Another limitation is the disproportionate inclusion of studies using the NPT. As this review sought to give an overview of the available approaches and their use, attempts were made to balance results where possible by providing narrative examples and descriptions for all sustainability approaches throughout the manuscript.

Finally, it is also important to note that this work does not seek to critique the included approaches but instead to consolidate the reported evidence on their use. Many of the sustainability approaches have been developed to encourage more systematic work and guide empirical studies; therefore, it should be acknowledged that this area of research is still in its infancy. Many of the conceptual frameworks and models aim to instigate knowledge generation on the determinants that influence sustainability processes and outcomes. In order to do so, they need to be applied and tested across real-world settings [4, 21, 116, 122]. Therefore, full insight into the impact of sustainability approaches will continue to be developed over time.

## Conclusion

This review provides healthcare practitioners and researchers with a consolidated summary of the current evidence base on sustainability approaches, including the benefits and challenges that arise through their use and the outcomes they have contributed to. The use of a sustainability approach can improve the knowledge of sustainability constructs, aid in analysis and increase focus to promote sustainability. They can also create challenges associated with their content, relevance and applicability. These findings highlight the need for thoughtful consideration of the potential impact prior to use. They also emphasise the remaining gaps in the literature, with a substantial amount of work still needed to ascertain the value and contributions of many sustainability approaches. In order to understand if these approaches are providing the foundations for rigorous inquiry and advancing sustainability research, well-planned studies recognising comprehensive sustainability outcome variables are needed.

## Supplementary information

**Additional file 1.** Sustainability approaches tracked in the literature.docx. Summarises the number of citations and full-text articles retrieved per approach.

**Additional file 2.** PRISMA screening fillable checklist for scoping reviews.Docx. The Preferred Reporting Items for Systematic reviews and Meta-Analyses extension for Scoping Reviews (PRISMA-ScR) checklist details the location of specific scoping review requirements.

**Additional file 3.** Quality assessment inclusion and exclusion of full text.docx Details on inclusions and exclusion of full text papers.

**Additional file 4.** Data extraction form with full data extraction for each included study

## Data Availability

All data generated or analysed during this study are included in this published article and its supplementary information files.

## Abbreviations

LoIn: Level of Institutionalisation Scale
NPT: Normalisation Process Theory
