# Supplementary material for: Making change last? Exploring the value of sustainability approaches in healthcare: a scoping review
Source: Health Res Policy Syst. 2020 Oct 13;18:120. doi: 10.1186/s12961-020-00601-0 (PMC7556957; doi:10.1186/s12961-020-00601-0)
Supplement: Supplementary file 1 — Additional file 1. Sustainability approaches tracked in the literature.docx. Summarises the number of citations and full-text articles retrieved per approach. [file 12961_2020_601_MOESM1_ESM.docx]

**Additional file 1: Sustainability Approaches Tracked in the Literature**

| Author | Sustainability Approach Name | Number of Citations | Full text retrieved | Articles Included in Review |
| --- | --- | --- | --- | --- |
| 1. Alexander, J.A. et al. | *The model for community health partnership sustainability* | 50 | 0 | 0 |
| 1. Amaya, A. et al. | *Conceptual framework for sustainability* | 4 | 0 | 0 |
| 1. Ament, S. et al. | *Strategies to sustain improvements in hospital practice* | 15 | 0 | 0 |
| 1. Atun, R. et al. | *A conceptual framework for analysing integration of targeted health interventions into health systems* | 115 | 1 | 1 |
| 1. Azeredo, B.T. , et al. | *Framework for investigating the sustainability of ARV provision* | 2 | 0 | 0 |
| 1. Blackford, J. & Street, A. | *The Advance Care Planning-Service Evaluation Tool (ACP-SET)* | 5 | 0 | 0 |
| 1. Blanchet, K. & Girois, S. | *The Sustainability Analysis Process (SAP)* | 8 | 1 | 1 |
| 1. Bray, P. et al. | *Sustainability Pyramid Model* | 38 | 0 | 0 |
| 1. Brinkenhoff & Goldsmith | *The analytical framework for Institutional sustainability* | 45 | 0 | 0 |
| 1. Chambers, D. et al. | *The Dynamic Sustainability Framework* | 172 | 1 | 0 |
| 1. Dauphinee, W. & Reznick, R. | *Framework for guiding change and managing and monitoring a successful multicentered network.* | 3 | 0 | 0 |
| 1. Dominick, G.M. et al. | *ENRICH Sustainability Survey* | 1 | 0 | 0 |
| 1. Dorsey, S. et al. | *NINR Logic Model for Center Sustainability* | 11 | 1 | 0 |
| 1. Edwards, J. C. et al. | *Catholic Healthcare partners HF-GAP Sustainability Assessment (AHRQ)* | 31 | 0 | 0 |
| 1. Feldstein, A.C. & Glasgow, R.E. | *Practical, Robust Implementation and Sustainability Model (PRISM)* | 245 | 1 | 0 |
| 1. Finch, T.L. et al. | *Technology Adoption Readiness Scale (TARS)* | 24 | 0 | 0 |
| 1. Fleiszer et al. | *Framework for the sustainability of healthcare innovations* | 12 | 1 | 1 |
| 1. Ford, J.H. et al. | *Strategies to Sustain Use of A-CHESS* | 11 | 0 | 0 |
| 1. Fox, A. et al. | *The sustainability of innovation theoretical framework* | 3 | 1 | 1 |
| 1. Goodman et al. | *Level of Institutionalisation (LoIn) Scale* | 95 | 7 | 5 |
| 1. Goodman, R. & Steckler, A. | *Model for Program Institutionalisation* | 275 | 0 | 0 |
| 1. Gruen, R.L. et al | *Model of health-programme sustainability* | 119 | 1 | 1 |
| 1. Hanson, D. et al. | *A systematic ecological framework to design sustainable interventions* | 15 | 0 | 0 |
| 1. Hodge L.M. & Turner, K.M.T. | *A Conceptual Framework of Supporting Factors* | 6 | 0 | 0 |
| 1. Isabalija, S.R. et al. | *Framework for e-medicine sustainability* | 17 | 0 | 0 |
| 1. Iwelunmor, Jet al. | *A conceptual framework* | 10 | 0 | 0 |
| 1. Johnson et al | *A Sustainability Planning Model* | 177 | 1 | 0 |
| 1. Knight, T. et al | *A framework for evaluating the sustainability of collaborative working* | 8 | 0 | 0 |
| 1. Leffers, J. & Mitchell, E. | *Conceptual Framework for Partnership and Sustainability in Global Health Nursing.* | 24 | 2 | 2 |
| 1. Lennox, L. et al. | *The Long Term Success Tool* | 3 | 0 | 0 |
| 1. Luke, D.A. | *Program Sustainability Assessment Tool (PSAT)* | 34 | 6 | 4 |
| 1. Maher, L. et al. | *NHS III Sustainability Model* | 4 | 3 | 3 |
| 1. Mancini, J.A. & Marek, L.I. | *Model of community-based program sustainability/Program Sustainability Index (PSI)* | 53 | 3 | 3 |
| 1. May, C. & Finch, T. | *Normalization Process Theory* | 354 | 19 | 30 |
| 1. May, C. et al | *Normalisation process Model* | 169 | 45 | 8 |
| 1. Melnyk & Fineout-Overholt | *The ARCC (Advancing Research and Clinical practice through close Collaboration) model* | 20 | 4 | 2 |
| 1. Nelson, D.E. et at. | *The five basic elements of program sustainability* | 11 | 0 | 0 |
| 1. Nystrom, M.E. et al | *Strategies to facilitate implementation and sustainability of large system transformations* | 8 | 0 | 0 |
| 1. Okeibunor, J. et al. | *A model for evaluating the sustainability of community-directed treatment with ivermectin* | 5 | 0 | 0 |
| 1. Olsen, I. T. | *Sustainability of health care: A framework for analysis* | 42 | 0 | 0 |
| 1. Parand, A. | *Strategies to sustain Safer Patient Initative (SPI)* | 14 | 0 | 0 |
| 1. Persaud, D | *The ELIAS (Enhancing Learning, Innovation, Adaptation, and Sustainability) Performance Management Framework* | 6 | 0 | 0 |
| 1. Racine, D.P. | *Model of sustaining innovations in their effectiveness* | 20 | 0 | 0 |
| 1. Rasschaert et al. | *Conceptual framework on sustainability of community-based programmes* | 13 | 0 | 0 |
| 1. Roy et el. | *Framework for Sustained Retention* | 6 | 0 | 0 |
| 1. Rudd, R. E. et al. | *A five-stage model for sustaining a community campaign* | 9 | 0 | 0 |
| 1. Sarriot, E.G. et al. | *Child Survival Sustainability Assessment (CSSA) framework and process* | 37 | 4 | 0 |
| 1. Sarriot, E.G. et al. Manual | *The Sustainability Framework* | 8 | 0 | 0 |
| 1. Saunders, R.P. | *LEAP Sustainability Assessment* | 13 | 0 | 0 |
| 1. Savaya, R. | *Projected Likelihood of Project’s Continuation* | 8 | 0 | 0 |
| 1. Schalock, R. et al. | *Sustainability model* | 7 | 0 | 0 |
| 1. Scheirer and Dearing | *Conceptual framework for sustainability of public health programs* | 152 | 2 | 1 |
| 1. Schell, S.F. et al. | *Capacity for sustainability framework* | 94 | 1 | 1 |
| 1. Shediac-Rizkallah & Bone | *Conceptual framework for planning for sustainability of community based health programs* | 369 | 2 | 2 |
| 1. Shigayeva, A. & Coker, R. | *Conceptual framework to support analyses of sustainability of communicable disease programmes* | 10 | 0 | 0 |
| 1. Sivaram, S. & Celentano, D.D. | *Conceptual framework to develop a strategy that will facilitate sustainability* | 13 | 0 | 0 |
| 1. Slaghuis, S.S. et al. | *A framework and a measurement instrument for sustainability of work practices in long-term care* | 25 | 2 | 2 |
| 1. Song, B. et al. | *The framework for sustainability evaluation of Community based LTC programmes* | 9 | 0 | 0 |
| 1. Sridharan, S. et al. | *Analysis of strategic plans to assess planning for sustainability of comprehensive community initiatives* | 18 | 0 | 0 |
| 1. Stefanini, A. & Ruck, N. | *Conceptual framework to monitor the performance of externally-assisted health projects* | 19 | 0 | 0 |
| 1. Story et al. | *Conceptual framework for the institutionalization of community-focused maternal, newborn and child health strategies into government health systems* | 8 | 0 | 0 |
| 1. Tuyet Hanh, T.T. et al. | *Framework for Evaluating the Sustainability of Community-based Dengue Control Projects* | 17 | 0 | 0 |
| Total |  | 3119 | 109 | 68 |
