# Supplementary material for: Making change last? Exploring the value of sustainability approaches in healthcare: a scoping review
Source: Health Res Policy Syst. 2020 Oct 13;18:120. doi: 10.1186/s12961-020-00601-0 (PMC7556957; doi:10.1186/s12961-020-00601-0)
Supplement: Supplementary file 3 — Additional file 3. Quality assessment inclusion and exclusion of full text.docx Details on inclusions and exclusion of full text papers. [file 12961_2020_601_MOESM3_ESM.docx]

| Author | Year | Method Name | Quality Assessment screening (for all types): 1. Are there clear research questions? YES, NO, CAN'T TELL | Quality Assessment screening (for all types): 2. Do the collected data allow to address the research questions? YES, NO, CAN'T TELL | Inclusion and Comments |
| --- | --- | --- | --- | --- | --- |
| 1. Ahmad M.S. & Abu Talib N.B. | 2015 | Program Sustainability Index (PSI) | Yes | Yes | Yes |
| 1. Ahmad M.S. & Abu Talib N.B. | 2016 | Program Sustainability Index (PSI) | Yes | Yes | Yes |
| 1. Atkins, S. et al. | 2011 | Normalisation process model | yes | Yes | Yes |
| 1. Bamford, C. et al. | 2012 | Normalisation process theory | Yes | Yes | Yes |
| 1. Blakeman et al. | 2012 | Normalisation process theory | Yes | Yes | Yes |
| 1. Blanchet K et al. | 2014 | The Sustainability Analysis Process (SAP) | Yes | Yes | Yes |
| 1. Bocoum et al. | 2017 | Normalisation process model | Yes | Yes | Yes |
| 1. Burau et al. | 2018 | Normalisation process theory | Yes | Yes | Yes |
| 1. Campbell, S. et al | 2011 | Gruen's Model of health-programme sustainability | Yes | Yes | Yes |
| 1. Chilundo et al. | 2015 | Shell's Capacity for sustainability framework | Yes | Yes | Yes |
| 1. Coupe, N. et al. | 2014 | Normalisation process theory | Yes | Yes | Yes |
| 1. Cramm, J.M. & Nieboer, A.P. | 2014 | Slaghuis's Framework and Instrument for sustainability | Yes | Yes | Yes |
| 1. Cramm, J.M. et al. | 2013 | Slaghuis's Framework and Instrument for sustainability | Yes | Yes | Yes |
| 1. Deconinck et al. | 2016 | Atun's Conceptual Framework for analysing integration of targeted health interventions into health systems | Yes | Yes | Yes |
| 1. Desveaux et al. | 2017 | Normalisation process theory | Yes | Yes | Yes |
| 1. Diaz del Castillo | 2017 | Conceptual framework for planning for sustainability of community-based health programs | Yes | Yes | Yes |
| 1. Dickinson et al. | 2017 | Normalisation process theory | Yes | Yes | Yes |
| 1. Doyle, C. et al. | 2013 | NHS III Sustainability Model | Yes | Can’t tell | Yes |
| 1. Drew, S. et al. | 2015 | Normalisation process theory | Yes | Yes | Yes |
| 1. Dugdale et al. | 2017 | Normalisation process model | Yes | Yes | Yes |
| 1. Farr et al. | 2018 | Normalisation process theory | Yes | Yes | Yes |
| 1. Fleiszer, A. et al | 2016 | Fleiszer's Framework for the sustainability of healthcare innovations | Yes | Yes | Yes |
| 1. Ford, J.H. et al. | 2014 | NHS III Sustainability Model | Yes | Yes | Yes |
| 1. Fox et al. | 2017 | Fox's sustainability of innovation theoretical framework | Yes | Yes | Yes |
| 1. Franx et al. | 2012 | Normalisation process theory | Yes | Yes | Yes |
| 1. Furler et al. | 2011 | Normalisation process model | Yes | Yes | Yes |
| 1. Gask et al. | 2010 | Normalisation process model | Yes | Yes | Yes |
| 1. Gillespie et al. | 2018 | Normalisation process theory | Yes | Yes | Yes |
| 1. Glynn et al. | 2018 | Normalisation process theory | Yes | Yes | Yes |
| 1. Godden & King | 2011 | Normalisation process model | Yes | Yes | Yes |
| 1. Green A.E. et al. | 2016 | Program Sustainability Index (PSI) | Yes | Yes | Yes |
| 1. Herbert et al. | 2017 | Normalisation process theory | Yes | Yes | Yes |
| 1. Higuchi, K.S. et al. | 2013 | NHS III Sustainability Model | Yes | Can’t tell | Yes |
| 1. Hooker, L. et al. | 2015 | Normalisation process theory | Yes | Yes | Yes |
| 1. Ibrahim et al. | 2018 | Normalisation process theory | Yes | Yes | Yes |
| 1. Johnson et al. | 2017 | Normalisation process theory | Yes | Yes | Yes |
| 1. Kennedy et al. | 2010 | Normalisation process theory | Yes | Yes | Yes |
| 1. Latter et al. | 2018 | Normalisation process theory | Yes | Yes | Yes |
| 1. Leon, N. et al. | 2013 | Normalisation process model | Yes | Yes | Yes |
| 1. Levin et al. | 2011 | The ARCC (Advancing Research and Clinical practice through close Collaboration) model | YES | YES | Yes |
| 1. Lloyd, A. et al. | 2013 | Normalisation process theory | yes | Yes | Yes |
| 1. Mair et al. | 2008 | Normalisation process model | Yes | Yes | Yes |
| 1. May et al. | 2011 | Normalisation process theory | YES | YES | Yes |
| 1. Moreland-Russel et al. | 2018 | Program Sustainability Assessment Tool (PSAT) | YES | YES | Yes |
| 1. Murray et al. | 2011 | Normalisation process theory | Yes | YES | Yes |
| 1. Naldemirci et al | 2017 | Normalisation process theory | Yes | YES | Yes |
| 1. O'Donnell and Kaner | 2017 | Normalisation process theory | YES | YES | Yes |
| 1. O'Donnell et al. | 2017 | Normalisation process theory | Yes | Yes | Yes |
| 1. Pentecost et al. | 2017 | Normalisation process theory | Yes | Yes | Yes |
| 1. Redman | 1997 | LoIn Scale | Yes | Yes | Yes |
| 1. Sanders et al. | 2011 | Normalisation process theory | Yes | Yes | Yes |
| 1. Scott et al. | 2018 | Conceptual framework for sustainability of public health programs. | Yes | Yes | Yes |
| 1. Scudder et al. | 2017 | Program Sustainability Assessment Tool (PSAT) | Yes | Yes | Yes |
| 1. Smith et al. | 2018 | Program Sustainability Assessment Tool (PSAT) | Yes | Yes | Yes |
| 1. Stoll et al. | 2015 | Program Sustainability Assessment Tool (PSAT) | Yes | Yes | Yes |
| 1. Stolldorf et al. | 2016 | LoIn Scale | Yes | Yes | Yes |
| 1. Sutton et al. | 2018 | Normalisation process theory | Yes | Yes | Yes |
| 1. Thomas, L.H. et al. | 2014 | Normalisation process theory | Yes | Yes | Yes |
| 1. Toledo Romanib et al. | 2007 | LoIn Scale | Yes | Can’t tell | Yes |
| 1. Trietsch, J. et al. | 2014 | Normalisation process theory | Yes | Yes | Yes |
| 1. Underwood, M.N. et al. | 2016 | Leffer's Conceptual Framework for Partnership and Sustainability | Yes | Yes | Yes |
| 1. Upvall et al. | 2018 | Leffer's Conceptual Framework for Partnership and Sustainability | Yes | Yes | Yes |
| 1. Van Acker et al. | 2012 | LoIn Scale | Yes | Yes | Yes |
| 1. Volker et al. | 2018 | Normalisation process theory | Yes | Yes | Yes |
| 1. Walker et al. | 2017 | Normalisation process theory | Yes | Yes | Yes |
| 1. Wallen et al. | 2010 | The ARCC (Advancing Research and Clinical practice through close Collaboration) model | Yes | Yes | Yes |
| 1. Winterton and Chambers | 2017 | Conceptual framework for planning for sustainability of community-based health programs | Yes | Yes | Yes |
| 1. Zakumumpa et al. | 2016 | LoIn Scale | Yes | Yes | Yes |
| 1. Aberg et al | 2017 | Normalisation process theory | Can't tell | Yes | No: unclear use |
| 1. Bamford, C. et al. | 2014 | Normalisation process theory | Can't tell | No | No: Methodology needs clarity and may not have addressed components explored in this ethnography |
| 1. Beck et al. | 2009 | PRISM (Practical, Robust Implementation and Sustainability Model) | No | Can’t tell | No: Does not meet criteria |
| 1. Bridges et al. | 2017 | Normalisation process theory | No | Can't tell | No: Does not meet criteria |
| 1. Calhoun, A. et al. | 2014 | Program Sustainability Assessment Tool (PSAT) | can't tell | Can't tell | No: Does not meet criteria |
| 1. de Brun, T. et al. | 2015 | Normalisation process theory | n/a | n/a | No: A systematic review |
| 1. Ehrlichet al. | 2012 | Normalisation process model | Yes | Can't tell | No: Does not meet criteria |
| 1. Elwyn et al. | 2008 | Normalisation process model | Can't tell | Can't tell | No: Does not meet criteria |
| 1. Forster, D.A. et al. | 2011 | Normalisation process model | yes | yes | No: Does not meet criteria on approach use |
| 1. Gallacher, K. et al. | 2011 | Normalisation process theory | No | Can't tell | No: Used for analysis purposes with little reference to sustainability |
| 1. Gask et al. | 2008 |  | Can't tell | Can't tell | No:The Study is more an analysis of another study. Therefore not applicable. |
| 1. Grant et al. | 2017 | Normalisation process theory | Yes | Can't tell | No: Need more clarification on use |
| 1. Gunn et al | 2010 | Normalisation process theory | Yes | can't tell | No: Does not meet criteria |
| 1. Jacobs, B. et al. | 2007 | Sarriot's Sustainability Framework (CSSA) | No | Can't tell | No: Does not meet criteria |
| 1. James, D.M. | 2011 | Normalisation process theory | No | Can't tell | No: Systematic review - not to be used |
| 1. Klinga et al. | 2018 | The Dynamic Sustainability Framework | No | Yes | No: Does not meet criteria |
| 1. Lees et al. | 2017 | NINR Logic Model | Yes | Can't tell | No: Does not meet criteria |
| 1. MacFarlane andO’Reilly-de Brún | 2012 | Normalisation process model | No | Yes | No: No sustainability discussion |
| 1. Mair et al. | 2012 | Normalisation process theory | n/a | n/a | No: Systematic review - not to be used |
| 1. May, C. et al. | 2007 | Normalisation process model | no | Can't tell | No: Does not meet criteria |
| 1. McGee-Lennon | 2017 | Normalisation process theory | YES | Can't tell | No: Does not meet criteria |
| 1. Mckinnon and Finch | 2018 | Normalisation process theory | No | No | No: Does not meet criteria |
| 1. Melnyk, B. | 2012 | The ARCC (Advancing Research and Clinical practice through close Collaboration) model | No | No | No: Does not meet criteria |
| 1. Melnyk, B. et al. | 2011 | The ARCC (Advancing Research and Clinical practice through close Collaboration) model | No | Can't tell | No: Does not meet criteria |
| 1. Morriss | 2008 | Normalisation process model | No | No | No: Does not meet criteria |
| 1. Moore et al. | 2017 | Conceptual framework for planning for sustainability of community based health programs | n/a | n/a | No: literature review |
| 1. Murray et al. | 2010 | Normalisation process model | Can't tell | Can't tell | No: Not an empirical study |
| 1. Papadakaki et al. | 2017 | Normalisation process theory | Yes | Can't tell | No: Clarification on methods needed |
| 1. Polus, B. et el. | 2012 | Normalisation process model | Can't tell | Yes | No: does not meet MMAT requirements |
| 1. Pope, C. et al. | 2013 | Normalisation process theory | Can't tell | Yes | No: Does not meet criteria |
| 1. Sarriot, E. et al. | 2009 | Sarriot's Sustainability Framework (CSSA) | Yes | Can’t tell | No: does not meet MMAT requirements |
| 1. Sarriot, E. et al. | 2014 | Sarriot's Sustainability Framework (CSSA) | Can't tell | Can’t tell | No: According to the MMAT, does not pass the two first questions |
| 1. Sarriot, E. et al. | 2015 | Sarriot's Sustainability Framework (CSSA) | n/a | n/a | No: Unable to retrieve full text. |
| 1. Scherier et al. | 2017 | Conceptual framework for sustainability of public health programs. | Yes | Can’t tell | No: Uncertainty of chosen sustainability approach |
| 1. Seppey et al. | 2017 | A sustainability planning model | Yes | Can't tell | No: Does not meet criteria |
| 1. Spargaro et al. | 2011 | Normalisation process theory | n/a | n/a | No: Unable to locate |
| 1. Tabak, R.G. | 2016 | Program Sustainability Assessment Tool (PSAT) | Yes | Yes | No: Actual use of a tool as a measurement was not done |
| 1. Watson et al. | 2011 | Normalisation process theory | Yes | Can't tell | No: scoping review |
| 1. Whitelaw et al. | 2017 | Normalisation process model | Yes | Yes | No: Does not meet criteria |
| 1. Wilkes & Rubin | 2009 | Normalisation process model | No | Can't tell | No: Does not meet criteria |
| 1. Wilson et al. | 2008 | LoIn Scale | Can’t tell | Can’t tell | No: Does not meet criteria |
